# Effect of intensive application of self-assembling peptide P11-4 with fluoride, casein phosphopeptide amorphous calcium phosphate fluoride and sodium fluoride on streptococcus mutans level in preschool children: a randomized controlled clinical trial

**DOI:** 10.1007/s00784-024-06133-z

**Published:** 2025-03-19

**Authors:** Sarah M. Khairy, Dalia M. Talaat, Sara A. M. Essa, Karin M. L. Dowidar

**Affiliations:** 1 Department of Pediatric Dentistry and Dental Public Health, Faculty of Dentistry, Alexandria University, Alexandria, Egypt; 2 Lecturer of Medical Microbiology and Immunology, Department of Medical Microbiology and Immunology, Faculty of Medicine, Alexandria University, Alexandria, Egypt; 3 Faculty of Dentistry, Alexandria University, Alexandria, Egypt

**Keywords:** Self-assembling peptide P11-4, Curodont repair fluoride plus, Sodium fluoride varnish, CPP-ACPF, Streptococcus mutans

## Abstract

**Objectives:**

To compare the effect of self-assembling peptide P11-4 with fluoride, casein phosphopeptide amorphous calcium phosphate fluoride (CPP-ACPF) varnish and 5% sodium fluoride varnish (NaF), on Streptococcus mutans (S. mutans) in dental plaque of preschoolers in addition to assessing change in plaque index after their intensive application.

**Methods:**

Sixty-six preschoolers were randomly assigned into three groups to receive triple applications of P11-4 with fluoride, CPP-ACPF varnish or NaF. S. mutans count in supragingival plaque samples was assessed at baseline (T0) and after the third application by 48 h (T1), one month (T2) and 3 months (T3). Multivariable linear regression compared the effect of these materials on S. mutans log count at various time intervals. Baseline plaque index was compared to that at T2 and T3.

**Results:**

All study groups presented a significant decrease in S. mutans count at T1, T2 and T3. Multivariable linear regression demonstrated a significant more reduction in S. mutans count in CPP-ACPF and NaF groups when compared to P11-4 with fluoride at T1. At T2 and T3, CPP-ACPF showed superior reduction in bacterial count than NaF and P11-4 with fluoride, with no significant difference between the latter two materials. Plaque index was significantly reduced at all study groups at T2 and T3, with CPP-ACPF being the most proficient.

**Conclusion:**

CPP-ACPF presented superior antibacterial effect when compared to P11-4 with fluoride or NaF which exhibited comparable antibacterial effect.

**Clinical relevance:**

Different remineralizing agents can have additive antibacterial effect against S. mutans that affects individual’s future caries experience.

## Introduction

Streptococcus mutans (S. mutans) has been proven to be a direct etiological factor of dental caries [1]. The level of S. mutans is considered an indicator of the individual’s caries experience [2]. A high count of S. mutans in dental plaque was found to be associated with increased risk of caries experience [3]. Many topical remineralizing agents can be applied to interrupt dental caries progression with additional antibacterial effect against S. mutans [4].

Professionally applied 5% sodium fluoride varnish (NaF) is considered the gold standard for dental caries prevention and remineralization of early carious lesions [5, 6]. NaF has been found to possess an antibacterial action against S. mutans [7, 8]. It is derived from the ability of fluoride to directly inhibit some cellular enzymes such as enolase enzyme, which is responsible for metabolizing carbohydrates and producing ATP for cellular maintenance and growth [4]. In addition, fluoride application can enrich healthier dental plaque microbiota [9].

With the advent of calcium phosphate-based remineralization systems, casein phosphopeptide amorphous calcium phosphate fluoride (CPP-ACPF) has proved excellent remineralization properties with additional antibacterial effect against S.mutans [6, 7]. Casein is considered the primary phosphopeptide which is found in milk [4]. Casein stabilizes the thermodynamically unstable ACP [4]. In addition, it has a pH buffering effect against acidic attacks of cariogenic bacteria. Moreover, the affinity of CPP-ACPF to bind to calcium was found to be twice that of bacterial cells, producing an extracellular free calcium concentration with resulting bacteriostatic or bactericidal effects [7]. Chandak et al. in 2016 [7] demonstrated that single application of CPP-ACPF complex significantly reduced S. mutans found in dental plaque of preschool children after 24 h. Moreover, Erkman and Oba in 2020 [8], found that a single application of CPP-ACPF varnish resulted in a significant decrease in S. mutans count found in the saliva of preschool children after one month. However, this antibacterial effect did not last at 3 months follow-up.

Intensive protocols of remineralizing agents’ application have been adopted in several studies with resulting superior remineralization effect and enhancement of antibacterial action when compared to single application [10–12]. Widyarman et al. in 2021 [13] reported that the intensive application of CPP-ACPF varnish in children changed their dental plaque microbial composition towards harboring healthier bacterial strains.

In an attempt to increase the antimicrobial action against S. mutans, self-assembling peptides containing tryptophan and arginine amino acids have been introduced. These peptides are classified as broad-spectrum antimicrobial peptides (AMPs) which can affect several pathogenic species such as bacteria, viruses and fungi [14, 15]. These peptides typically form amphipathic structures while contacting the bacterial cell membrane, affecting its microleakage, intracellular DNA synthesis, and other enzymatic activities, including those responsible for protein production [14, 15]. Self-assembling peptide P11-4 (Curodont™ repair) has been introduced as a biomimetic enamel remineralizing agent with potential antimicrobial action against S. mutans [16]. It is an oligomeric peptide that is composed of 11 amino acids at the sequence of Ace-Gln-Gln-Arg-PheGlu-Trp-Glu-Phe-Glu-Gln–Gln-NH2, which includes both arginine and tryptophan [17]. In an invitro study by Gayas et al. in 2023 [18], the antibacterial effect of P11-4, in the form of a gel, against S. mutans was found to be significantly superior to that of fluoride-enhanced hydroxyapatite gel, acidulated phosphate fluoride, and chlorohexidine gluconate gel.

Curodont™ Repair Fluoride Plus is a new product that contains P11-4 with a small dose of sodium fluoride (0.05% NaF/ 500 ppm fluoride). In an invitro study by Elkaddah et al. in 2024 [19], the antimicrobial efficacy of P11-4 with fluoride presented significantly better antimicrobial action against S. mutans when compared to 5% NaF Varnish.

Self-assembling peptide P11-4 with fluoride, CPP-ACPF and NaF are different remineralizing agents with additional antibacterial effect against S. mutans which can be achieved through different mechanisms of action [7, 16]. There is insufficient evidence in the literature comparing the antibacterial effect of intensive application of P11-4 with fluoride versus CPP-ACPF when compared to that of 5% NaF. That’s why, the current study aimed to assess and compare the effect of intensive application of P11-4 with fluoride, CPP-ACPF varnish, and 5% NaF varnish on S. mutans level found in dental plaque of preschool children. The null hypothesis was that there would be no difference in the antibacterial effect of the three agents against S. mutans when applied in an intensive mode to primary teeth of preschool children.

## Methods

### Trial design and settings

This study was a three parallel-arm randomized clinical trial that was set up and reported according to the CONSORT guidelines [20]. The clinical part of the study was done in the clinic of the Pediatric Dentistry Department in the Faculty of Dentistry, Alexandria University, and microbiological samples were analyzed in the microbiology lab, Faculty of Medicine, Alexandria University, Egypt.

### Ethical considerations

Ethical approval was obtained from the Research Ethics Committee, Faculty of Dentistry, Alexandria University (IRB No. 001056 – IORG 0008839). The study was conducted in accordance with the World Medical Association Declaration of Helsinki [21]. The trial was registered at ClinicalTrials.gov NCT06566833. An informed consent was signed by each child parent/guardian after discussing the aim of the study, methodology, periodicity of recalls, benefits and side effects of the materials used.

Oral hygiene instructions and dietary guidance were discussed with each child and his/her guardian, prior to the beginning of the study and at each follow up. These included brushing teeth at least twice daily, especially at bed time, using a pea-sized fluoridated tooth paste using horizontal scrubbing technique, and flossing once daily if proximal contacts are closed [22].

### Sample size estimation

Sample size was estimated assuming 5% alpha error and 80% study power. The Mean ± SD of S. mutans count (CFU/ml) reported by Patel et al. [12], three months after intensive application of CPP-ACPF varnish was reported to be 13.83 ± 5.81 while it was 23 ± 9.67 after intensive application of NaF varnish. The minimum sample size was calculated to be 13 patients per group, which was increased by 20% to be 15 patients per group, considering loss to follow up. The minimum total sample size = number per group x number of groups = 15 × 3 = 45 patients. Sample size was based on Rosner’s method [23] calculated by G power 3.0.10 [24].

### Eligibility criteria

Sixty-six healthy children aged 3–6 years who had primary dentition with at least two active WSLs (according to ICDAS-II criteria [25]) were enrolled in the study. Children were excluded if they received treatment that can reduce salivary flow, administrated antibiotics within one month prior to beginning the study [26], received fluoride varnish in a period < 3 months before commencing the study or if they had proven or suspected milk protein allergy [27].

### Randomization and allocation concealment

Subjects fulfilling the eligibility criteria were randomly assigned by a computer-generated random allocation sequence into 3 groups in blocks of sixth with an allocation ratio of 1:1:1. Test group I (*n* = 22) received intensive application of P11-4 with 0.05% NaF (Curodont™ Repair Fluoride Plus, ^V^VARDIS. PROFESSIONAL, Switzerland), test group II (*n* = 22) received intensive application of 2% CPP-ACP with 5% sodium fluoride (MI varnish^®^, GC GC America Inc., USA), and group III (control; group, *n* = 22 patients) received intensive application of 5% NaF (Duraphat^®^ varnish, Colgate, Palmolive Co., New York., USA).

To establish allocation concealment, each participant’s guardian received a serial number for his/her child’s allocation. A duplicate of this number was kept in an opaque envelope that was drawn to determine the assignment of the patient only at the time of the intervention by a trial independent person.

### Blinding

This study was triple-blinded, as the participants, the investigator, who was responsible for counting bacterial colonies, and the statistician were blinded to the group divisions. Since the method of application of P11-4 with fluoride differs from that of CPP-ACPF varnish and NaF varnish, and the form of the commercial products differ, blinding of the primary investigator was not possible.

### Intervention

Children completed dental treatment for cavitated carious lesions prior to the beginning of the study to minimize confounders and to be able to assess the actual antibacterial effect of the tested materials on S. mutans count [8, 28]. This was followed by a washout period of ten days to avoid the effect of any restorative material on S. mutans count [12].

Baseline assessment of caries experience by decayed, missed, and filled teeth (dmft) index [29], caries risk assessment score by CAMBRA [30], and baseline plaque index score using modified Sillness and Loe plaque index [31] were recorded prior to plaque sampling.

### Baseline plaque sample collection

The selected children were asked not to brush their teeth in the morning of plaque sample collection and were refrained from eating or drinking anything other than water 1–2 h before this procedure [7, 26]. Pooled supragingival plaque samples were collected from the labial and lingual surfaces of the teeth affected with active WSLs by sterilized dental toothpicks [28]. Each sample was kept immediately in an Eppendorf tube (1.5 ml) containing 0.5 ml of sterile brain heart infusion (BHI) broth media and transported within 30 min to the microbiological lab.

### Remineralizing agents’ application

Dental prophylaxis with a brush without a paste was done before remineralizing agents’ application that was applied on the teeth affected with WSLs according to the manufacturer’s instructions. P11-4 with fluoride was applied after surface treatment by applying 2% sodium hypochlorite for 20 s for removal of organic pellicle, and then by applying 35% phosphoric acid for 20 s for removal of inorganic deposits. Afterwards, the surface was rinsed with water, dried, and the material was applied by squeezing the applicator sponge above the lesion for 5 min [32]. Both CPP-ACPF varnish and NaF varnish were applied after partial isolation, by using an applicator, on the affected dried areas [33, 34].

The patients were asked to refrain from eating, drinking, or rinsing their mouths for at least 30 min after P11-4 with fluoride application and for 4 h after CPP-ACPF varnish and NaF varnish application. Two more applications of each material took place after two and four weeks from baseline [11] using the same method of application and the patients were asked to follow the same post application instructions.

### Microbiological assessment procedure

For culturing S. mutans, all samples were dispersed, at the microbiological lab, by vortexing for 30 s, then diluted in decimal (1:10) series from 10 − 1 to 10 − 4 in sterile phosphate-buffered saline at a pH of 7.2. Aliquots of each dilution were incubated into sterile plates containing freshly prepared Mitis Salivarius Agar (MSA) supplemented with 300 units of Bacitracin and 20% sucrose (mitis salivarius‑bacitracin agar), which was used for the isolation of oral streptococci. Mitis Salivarius Agar plates were anaerobically incubated at 37 degrees Celsius for 48 h using a gas pack system before colony counting [7].

Following the predetermined incubation periods, S. mutans were identified on the basis of their characteristic morphological appearance on Mitis Salivarius Agar plates (frosted glass appearance) [7]. Identification of S. mutans was confirmed by film morphology (gram-positive cocci), Catalase test (negative), and bile test (negative). The number of bacterial colonies was counted by a single investigator. The total number of colonies was determined and expressed as colony forming units (CFUs) using the following equation:

Number of colonies/mL (CFU/mL) = Number of counted colonies × the dilution factor (1000)/ Volume in ml (0.5).

### Study outcomes

The outcome measure of the present study was done by assessing the change in S. mutans count found in supragingival plaque samples at baseline (T0), and then after the third application by 48 h (T1), one month (T2) and three months (T3). Post-application plaque sampling was done using the same method as previously discussed at baseline. In addition, the change in plaque index as determined by Sillness and Loe plaque index [31] was assessed after 1 month (T2) and after 3 months (T3).

### Statistical analysis

Data were statistically analyzed using SPSS software 23.0 (SPSS Inc., Chicago, IL, USA). All tests were two-tailed, and the level of significance used was set at *p* value < 0.05. Intention to treat analysis was done for cases lost to follow-up. Normality of quantitative variables was checked using Kolmogorov-Smirnov test and Q-Q plots. Normal distribution of all variables was confirmed, except the plaque index score. One-way ANOVA and Kruskal Wallis tests were employed for inter-group comparison, followed by Tukey’s and Dunn’s post hoc tests, respectively, with Bonferroni correction to adjust for type I error. Repeated Measures ANOVA and Friedman tests were used to analyze changes in bacterial log count and plaque index across follow up intervals. Multivariable linear regression model was developed to assess the antibacterial effect of P11-4 with fluoride (Test 1) and CPP-ACPF (Test 2) as compared to NaF (control) on bacterial log count at T1, T2, and T3. The model was adjusted for age, sex, dmft, and caries risk score in addition to baseline plaque index, and S. mutans count at baseline as confounders.

## Results

**Fig. 1:**
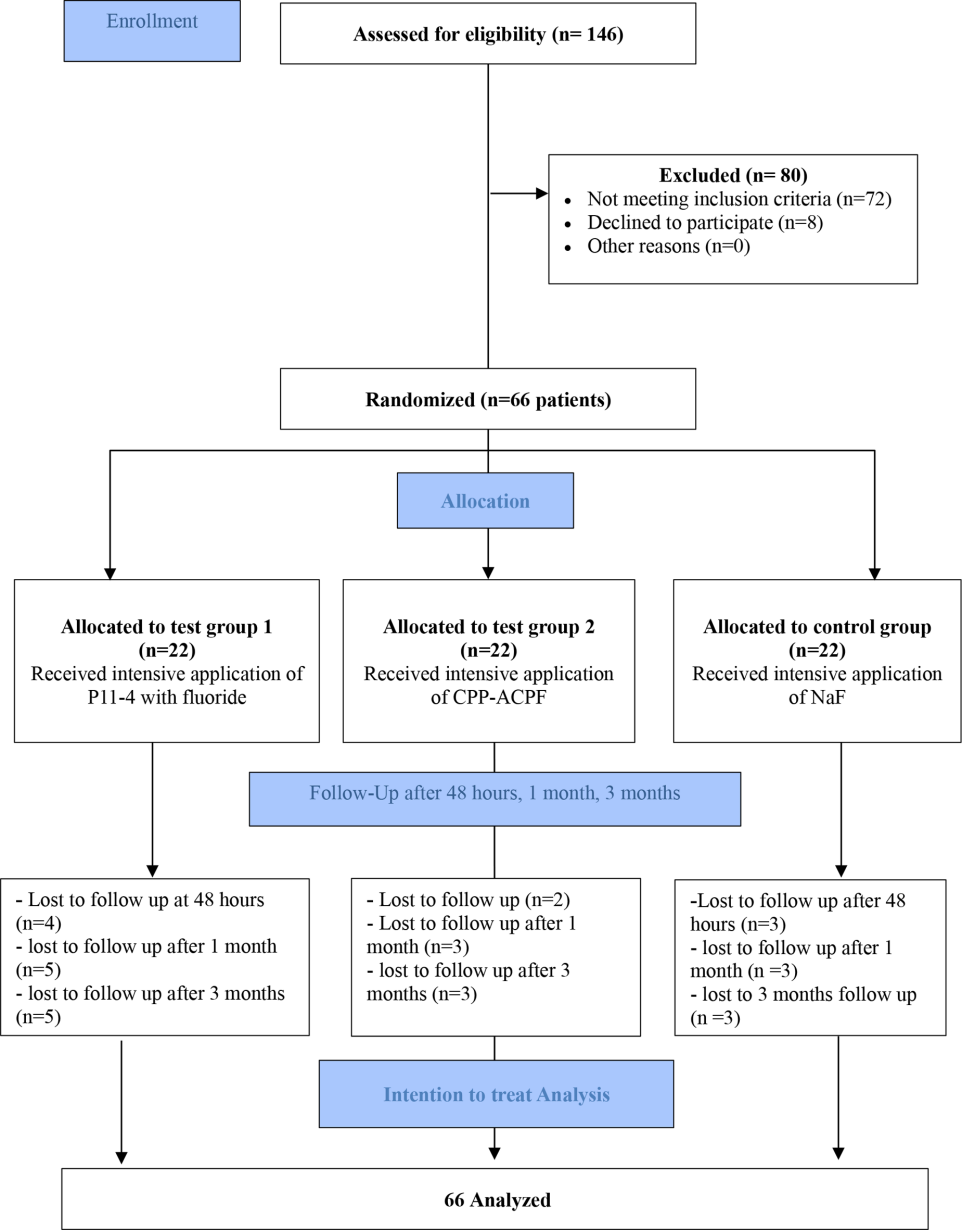
Consort flowchart of the current study

Among 146 children assessed for eligibility, a total of sixty-six preschool children were included in the current study and randomized to one of the three study groups (Fig. 1). Recruitment of study participants started in August 2023, application of materials ended at October 2023, and then follow-up lasted till January 2024. Demographic data presentation (age, gender) and baseline oral characteristics (dmft score, CAMBRA score, baseline plaque index) of the study sample are described in Table 1. No statistically significant difference was observed in either of these parameters among the study groups.

**Table 1:** Baseline characteristics of the study sample

Variables		P11-4 with fluoride (*n* = 22 patients)	CPP-ACPF (*n* = 22 patients)	NaF (*n* = 22 patients)	*p* value
Age in years	Mean ± SD	4.45 ± 0.86	4.86 ± 0.83	5.00 ± 0.87	0.097
Gender: n (%)	Male	9 (40.9%)	9 (40.9%)	13 (59.1%)	0.378
	Female	13 (59.1%)	13 (59.1%)	9 (40.9%)	
dmft	Mean ± SD	7.14 ± 2.95	8.59 ± 2.15	8.82 ± 2.22	0.056
CAMBRA	Mean ± SD	10.09 ± 1.69	10.00 ± 1.72	10.55 ± 1.71	0.528
Plaque index	Median (IQR)	1.50 (0.47)	1.6 (0.20)	1.50 (0.36)	0.454
Number of WSLs	Median (IQR)	2 (2)	3 (1)	2 (2)	0.650

**Fig. 2:**
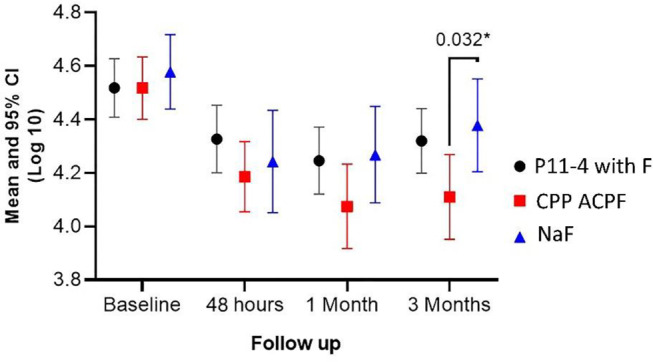
Mean and 95% CI of S. mutans log count in all study groups at all follow-up intervals

Intra-group comparison revealed a statistically significant reduction in S. mutans count in all three study groups at T1, T2, and T3 (*P* < 0.001), denoting a significant effect of the three materials against S. mutans that extended up to three months. Inter-group comparison revealed no statistically significant reduction in bacterial count among the three study groups at T1 (*P* = 0.369) and T2 (*P* = 0.133). However, at T3, there was a statistically significant difference among the groups (*P* < 0.028) with the greatest reduction presented in the group treated with CPP-ACPF. (Table 2) Pairwise comparison showed a statistically significant difference between the group treated with CPP-ACPF and that treated with NaF at T3 (*p* = 0.032) (Fig. 2).

**Table 2:** Comparison of total and log counts of S. mutans (CFU/ml) among the study groups

Follow Up		P11-4 with fluoride (*n* = 22 patients)	CPP-ACPF (*n* = 22 patients)	NaF (*n* = 22 patients)	*p* value^1^
		Mean ± SD			
Baseline (T0)	Total Count	3.67 ± 2.10 × 10^4^	3.82 ± 2.54 × 10^4^	4.28 ± 2.69 × 10^4^	0.701
	Log_10_	4.50 ± 0.25	4.50 ± 0.27	4.54 ± 0.31	
48 h (T1)	Total Count	2.48 ± 1.42 × 10^4^	1.88 ± 1.32 × 10^4^	2.38 ± 1.84 × 10^4^	0.369
	Log_10_	4.33 ± 0.27	4.19 ± 0.08	4.24 ± 0.38	
1 Month (T2)	Total Count	2.10 ± 1.43 × 10^4^	1.54 ± 1.09 × 10^4^	2.54 ± 2.18 × 10^4^	0.133
	Log_10_	4.25 ± 0.27	4.08 ± 0.34	4.27 ± 0.36	
3 Months (T3)	Total Count	2.47 ± 1.58 × 10^4^	1.67 ± 1.19 × 10^4^	3.16 ± 2.49 × 10^4^	0.028*
	Log_10_	4.32 ± 0.26^ab^	4.11 ± 0.34^a^	4.38 ± 0.35^b^	
*p value* ^*2*^		< 0.001*	< 0.001*	< 0.001*	
*Post hoc p values*		***p***^***1***^ < 0.001* ***p***^***2***^ < 0.001* ***p***^***3***^ < 0.001*	***p***^***1***^ < 0.001* ***p***^***2***^ < 0.001* ***p***^***3***^ < 0.001*	***p***^***1***^ < 0.001* ***p***^***2***^ < 0.001* ***p***^***3***^ < 0.001*	

* Statistically significant difference at *p* value < 0.05, *p* value^1^: One Way ANOVA Test, *p* value^2^: Repeated Measures ANOVA Test, Different superscript lowercase letters denote statistically significant difference between groups, *p*1: comparison between baseline and 48 h, *p*2: comparison between baseline and 1 month, *p*3: comparison between baseline and 3 months

There was a statistically significant reduction in plaque index score in all study groups at T2 and T3 (*P* < 0.001). Inter-group comparison revealed a statistically significant difference between the three study groups at T2 (*p* = 0.005) and T3 (*p* = 0.008). Pairwise comparison revealed a statistically significant difference between the group treated with CPP-ACPF in comparison to that treated with P11-4 with fluoride. However, there was no statistically significant difference between the group treated with P11-4 with fluoride and that treated with NaF or between the group treated with CPP-ACPF and that treated with NaF. (Table 3)

**Table 3:** Comparison of plaque index among the three study groups

Variables		P11-4 with fluoride (*n* = 22 patients)	CPP-ACPF (*n* = 22 patients)	NaF (*n* = 22 patients)	*p* value^1^
Baseline	Mean ± SD	1.63 ± 0.34	1.55 ± 0.19	1.52 ± 0.24	0.454
	Median (IQR)	1.50 (0.47)	1.60 (0.20)	1.50 (0.36)	
1 Month	Mean ± SD	1.40 ± 0.21	1.24 ± 0.16	1.33 ± 0.16	0.005*
	Median (IQR)	1.33^a^ (0.19)	1.24^b^ (0.17)	1.33^ab^ (0.19)	
3 Months	Mean ± SD	1.35 ± 0.19	1.27 ± 0.11	1.28 ± 0.19	0.008*
	Median (IQR)	1.37^a^ (0.3)	1.33^b^ (0.17)	1.33^ab^ (0.19)	
*p* ^2^		***p*** < 0.001*	***p*** < 0.001*	***p*** < 0.001*	
*Post hoc*		***p***^***1***^ < 0.001* ***p***^***2***^ < 0.001* *P*^*3*^ = 0.598	***p***^***1***^ < 0.001* ***p***^***2***^ < 0.001* *P*^*3*^ = 0.291	***p***^***1***^ < 0.001* ***p***^***2***^ < 0.001* *P*^*3*^ = 0.706	

* Statistically significant difference at p value < 0.05, *p* value^1^: Kruskal Wallis Test, *p* value^2^: Friedman Test, Different superscript lowercase letters denote statistically significant difference between groups. *p*1: comparison between baseline and 1 month, *p*2: comparison between baseline and 3 months, *p*3: comparison between 1 month and 3 months

When comparing the effect of P11-4 with fluoride versus that of NaF, the multivariable linear regression model revealed a statistically significant more reduction in bacterial log count in the NaF group at T1 (*P* < 0.001) with a reduction coefficient of 0.180. However, the antibacterial effect of P11-4 with fluoride increased by time to achieve a reduction coefficient of 0.087 and 0.010 at T2 and T3 respectively resulting in no statistically significant difference with that of NaF (*p* = 0.111 at T2, and *p* = 0.836 at T3). (Table 4)

When comparing the effect of CPP-ACPF versus that of NaF, the model demonstrated that at T1, the reduction coefficient of S. mutans log count was 0.009, with no statistically significant difference between both materials (*P* = 0.108). However, a statistically significant more reduction in bacterial log count was noted in the CPP-ACPF group at T2 (*P* = 0.008) and at T3 (*P* < 0.001) with a reduction coefficient of -0.130 and − 0.216 respectively. (Table 4)

**Table 4:** Multivariable linear regression assessing the effect of P11-4 with fluoride and CPP-ACPF when compared to NaF on bacterial log count at different follow up intervals

	48 h (T1)		1 month (T2)		3 months (T3)	
	B (95% CI)	*P* value	B (95% CI)	*P* value	B (95% CI)	*P* value
P11-4 with fluoride vs. NaF	0.180 (0.098, 0.261)	< 0.001*	0.087 (-0.020, 0.194)	0.111	0.010 (-0.085, 0.105)	0.836
CPP-ACPF vs. NaF	0.009 (-0.064, 0.082)	0.108	-0.130 (-0.226, -0.034)	0.008*	-0.216 (-0.301, -0.132)	< 0.001*

Model was adjusted for age, sex, dmft, and CAMBRA score, in addition to baseline plaque score, and baseline bacterial log count, *Statistically significant difference at *p* value < 0.05

## Discussion

Dental plaque biofilm is a microecological habitat that colonizes the tooth surface and facilitates the attachment of S. mutans [35]. The disruption of the micro-ecological balance of plaque bacteria towards harboring a high number of the acid producing and acid-resistant S. mutans precipitate to the onset of dental caries [4]. In addition, S. mutans count in preschoolers was found to be significantly correlated with ECC severity [36] and is considered a risk predictor for their future caries experience [37]. As a result, the contemporary strategy to prevent and control dental caries targets not only the prevention of cariogenic bacterial colonization, but also attempts to restore eubiosis of a healthy biofilm composition [4].

Intensive application of different remineralizing agents was found to achieve better remineralization as well as enhanced antibacterial action against S. mutans [11, 12]. That’s why, the present study adopted an intensive mode of application of different remineralizing agents with potential antibacterial effect with the aim of comparing the antibacterial effect of these materials against S. mutans.

The results of the present study rejected the null hypothesis. It demonstrated that, all study groups resulted in a significant reduction in S. mutans count at all time intervals and after statistical adjustment of cofounders, it was found that, after 48 hours, both NaF and CPP-ACPF presented a significant and comparable antibacterial effect against S. mutans, which was statistically more significant than P11-4 with fluoride. This could be attributed to the “*Burst Effect*” of fluoride-containing varnishes, which is a typical pattern of fluoride ion release from fluoride varnishes that contain 22,600 ppm fluoride, with the greatest amount being released within the first few days after application, followed by a quick decrease into lower levels afterwards [28]. On the contrary, P11-4 with fluoride contains much less amount of fluoride (only 500 ppm) that may not exhibit this ‘‘*Burst Effect*”. Moreover, self-assembling peptides undergo self-assembly into fibrillar scaffolds and the antibacterial efficacy of some antimicrobial peptides can be considerably enhanced upon assembly into nanostructures that may take few days [14]. This by its turn raises the concern of the need for better understanding of aggregation, assembly, and disassembly of self-assembling peptide P11-4.

Moreover, the results of the present study revealed that P11-4 with fluoride showed a comparable antibacterial effect to that of NaF against S. mutans after 1 month and 3 months. This antibacterial effect of P11-4 with fluoride could be due to the presence of tryptophan and arginine amino acids. Arginine has been found to possess a multimodal antimicrobial effect against S. mutans and that was claimed to be complementary to the mechanism of action of fluoride [38]. In addition, it can be metabolized by some strains of bacteria in dental plaque (arginolytic bacteria) with resulting production of ammonia that help increase plaque pH. Moreover, enriching arginolytic bacteria may help shifting dysbiotic dental plaque towards harboring less cariogenic bacteria (prebiotic effect) [15]. These results come in partial accordance with a recent study by Atteya et al. [16] who demonstrated a significant reduction in S. mutans count in subjects treated with P11-4 in comparison to those treated with NaF after one month of follow-up (*p* = 0.049). However, this difference was not significant after three-month. The notable antibacterial effect of P11-4 with fluoride was confirmed by an invitro study which was done by El-Kaddah et al. [19], who demonstrated the formation of inhibition zones on agar plates cultured with S. mutans and treated with P11-4 with fluoride.

In the present study, CPP-ACPF exhibited enhanced antibacterial effect against S. mutans when compared to P11-4 with fluoride or NaF varnish. These results come in accordance with those observed by Patel et al. [12] who compared the effect of intensive application of CPP-ACPF varnish against that of NaF varnish on children with mixed dentition. On the other hand, Erkmen and Oba A in 2020 [8], reported a significant reduction of S. mutans after a single application of either 5% NaF varnish or CPP-ACPF varnish to children with primary dentition, with no significant difference between the two materials. In addition, this antibacterial effect was reported to extend up to one month only which may indicate that the intensive mode of application is favorable for extended antibacterial action of these materials.

Moreover, the observed antibacterial effect of CPP-ACPF in the current study was supported by a notable reduction in plaque index as compared to NaF varnish or P11-4 with fluoride. A possible explanation is that CPP-ACP are nanocomplexes which are soluble in human saliva, with the ability to bind to the salivary pellicle, thus affecting the rate of formation of dental plaque and consequently the adhesion of S. mutans on the tooth surface [39, 40]. Moreover, the application of CPP-ACP in high concentrations was found to inhibit dental plaque biofilm formation by 90% after 4 h in addition to altering their morphology thus inhibiting the planktonic growth of these micro-organisms [40].

The limitations of the present study include the need to evaluate a longer-term effect of intensive application of the studied materials against S. mutans. In addition, the present study evaluated the antimicrobial effect of the studied materials against S. mutans only. That’s why future studies are recommended to evaluate the effect of these materials on other caries-associated microorganisms such as streptococcus sobrinus, lactobacilli, and candida albicans. Moreover, other culture-independent detection methods, such as rRNA sequencing, can be used to be able to detect changes in oral microecology in a more comprehensive way. Furthermore, future research should address the antimicrobial effect of the studied remineralizing agents when applied to children with mixed dentition who passed through the second window of infectivity, after the eruption of the first permanent molar.

## Conclusion

Within the limitations of the present study, it was concluded that intensive application of P11-4 with fluoride, CPP-ACPF, or NaF resulted in a significant reduction in S. mutans count as well as plaque index that extended up to 3 months with CPP-ACPF being the most proficient. P11-4 with fluoride can be considered a self-assembling peptide with potential antimicrobial activity.

## Data Availability

No datasets were generated or analysed during the current study.
